# p32 protein levels are integral to mitochondrial and endoplasmic reticulum morphology, cell metabolism and survival

**DOI:** 10.1042/BJ20121829

**Published:** 2013-07-12

**Authors:** MengJie Hu, Simon A. Crawford, Darren C. Henstridge, Ivan H. W. Ng, Esther J. H. Boey, Yuekang Xu, Mark A. Febbraio, David A. Jans, Marie A. Bogoyevitch

**Affiliations:** *Department of Biochemistry and Molecular Biology, Bio21 Molecular Science and Biotechnology Institute, University of Melbourne, Melbourne, VIC 3010, Australia; †School of Botany, University of Melbourne, Melbourne, VIC 3010, Australia; ‡Cellular and Molecular Metabolism Laboratory, Baker IDI Heart and Diabetes Institute, Melbourne, VIC 3004, Australia; §Department of Biochemistry and Molecular Biology, Monash University, Melbourne, VIC 3800, Australia

**Keywords:** endoplasmic reticulum (ER) morphology, mitochondrial metabolic activity, mitochondrial morphology, stress response, CCCP, carbonyl cyanide *m*-chlorophenylhydrazone, DMEM, Dulbecco’s modified Eagle’s medium, Drp-1, dynamin-related protein 1, ECAR, extracellular acidification rate, ER, endoplasmic reticulum, FCCP, carbonyl cyanide *p*-trifluoromethoxyphenylhydrazone, gC1qR, receptor for globular head domains complement 1q, JNK, c-Jun N-terminal kinase, LDH, lactate dehydrogenase, Mfn, mitofusin, OCR, oxygen consumption rate, Opa1, optical atrophy 1, p32 N_m_-terminus, N-terminus of mature p32 protein, PARP, poly(ADP-ribose) polymerase, PJNK, phosphorylated c-Jun N-terminal kinase, RIPA, radioimmunoprecipitation assay, TBST, 10 mM Tris/HCl, pH 7.5, 150 mM NaCl and 0.1% Tween 20, TEM, transmission electron microscopy, XTT, sodium 3′-[1-(phenylaminocarbonyl)-3,4-tetrazolium]-bis (4-methoxy-6-nitro) benzene sulfonic acid hydrate

## Abstract

p32 [also known as HABP1 (hyaluronan-binding protein 1), gC1qR (receptor for globular head domains complement 1q) or C1qbp (complement 1q-binding protein)] has been shown previously to have both mitochondrial and non-mitochondrial localization and functions. In the present study, we show for the first time that endogenous p32 protein is a mitochondrial protein in HeLa cells under control and stress conditions. In defining the impact of altering p32 levels in these cells, we demonstrate that the overexpression of p32 increased mitochondrial fibrils. Conversely, siRNA-mediated p32 knockdown enhanced mitochondrial fragmentation accompanied by a loss of detectable levels of the mitochondrial fusion mediator proteins Mfn (mitofusin) 1 and Mfn2. More detailed ultrastructure analysis by transmission electron microscopy revealed aberrant mitochondrial structures with less and/or fragmented cristae and reduced mitochondrial matrix density as well as more punctate ER (endoplasmic reticulum) with noticeable dissociation of their ribosomes. The analysis of mitochondrial bioenergetics showed significantly reduced capacities in basal respiration and oxidative ATP turnover following p32 depletion. Furthermore, siRNA-mediated p32 knockdown resulted in differential stress-dependent effects on cell death, with enhanced cell death observed in the presence of hyperosmotic stress or cisplatin treatment, but decreased cell death in the presence of arsenite. Taken together, our studies highlight the critical contributions of the p32 protein to the morphology of mitochondria and ER under normal cellular conditions, as well as important roles of the p32 protein in cellular metabolism and various stress responses.

## INTRODUCTION

p32 [also known as HABP1 (hyaluronan-binding protein 1), gC1qR (receptor for globular head domains complement 1q) or C1qbp (complement 1q-binding protein)], a conserved eukaryotic protein [1], was originally identified as a pre-mRNA splicing factor SF2-associated protein [2] as well as a globular protein capable of interacting with the complement component C1q [3]. Although p32 has been reported to exist in multiple subcellular compartments [4–6], several studies have demonstrated that p32 is predominantly localized in the mitochondria of various cell types [1,7,8]. Indeed, the N-terminus of the immature p32 protein includes a stretch of 73 amino acids that contains a mitochondrial localization sequence [3].

The possible functions of mitochondrial p32 have been studied previously in the context of disease pathogenesis, especially in the development of cancer [6,7]. The interaction with mitochondrial p32 has been shown to be required for the induction of mitochondrial-dependent cell death in cancer cells by the pro-death Bcl2 family protein Hrk [9] and the tumour suppressor human p14 ARF [8], suggesting that p32 potentially acts as a tumour suppressor. However, there have been multiple reports of increased p32 protein levels in various human cancer cell lines and carcinoma [6,10]. Indeed, previous studies using both gain- and loss-of-function approaches have demonstrated a pro-tumorigenic effect of mitochondrial p32 in breast cancer cells [11]. In agreement with this, investigations on tumour metabolism have shown that p32 knockdown cancer cells shift their metabolism from oxidative phosphorylation towards aerobic glycolysis, thus becoming poorly tumorigenic when p32 levels are lowered [10].

Previous studies have also reported the importance of the maintenance of mitochondria dynamics in energy metabolism and cell survival [12–14]. Mitochondria typically form a reticular network that is a dynamic interconnected system that undergoes constant cycles of mitochondrial fusion and fission [12–14]. The cellular architecture and the dynamic reorganization of the network are tightly controlled by a number of key proteins such as Drp-1 (dynamin-related protein 1), Opa1 (optical atrophy 1) and the Mfn (mitofusin) 1 and Mfn2 proteins, which are found participating in mitochondrial fusion/fission machinery as well as affecting the metabolism and regulation of cell death [15–17].

To examine the functional roles played by p32, we evaluated mitochondrial morphology and metabolic activity after modulating p32 expression levels in HeLa cells. This revealed striking changes in mitochondrial morphology, mitochondrial ATP turnover rate and basal respiration, and indicated for the first time the impact on ER (endoplasmic reticulum) structure, including a dissociation of ribosomes from the ER following p32 depletion. Significantly, we also observed a differential impact of p32 silencing on cell survival following exposure of cells to abiotic stress. Thus the loss of the p32 protein enhanced either cell death or cell survival, dependent on the nature of the stress. Taken together, the results of the present study reveal the critical importance for maintaining p32 levels to maintain appropriate mitochondrial and ER structures, and highlight the complexities of the contributions by altered p32 levels within the context of disease pathogenesis, particularly in the development of cancer and the response of these cells to abiotic stresses such as exposure to the chemotherapeutic agent cisplatin.

## EXPERIMENTAL

### Cell culture and stress treatments

HeLa cells were maintained in a humidified 5% CO_2_ atmosphere at 37°C in growth medium [DMEM (Dulbecco's modified Eagle's medium) containing L-glutamine (Gibco) and 4.5g/l D-glucose supplemented with 10% FBS (DKSH Australia) and 100 units/ml penicillin and streptomycin (Gibco)]. Cells were passaged at 3-day intervals by dissociation with trypsin-EDTA solution and then replating. For immunofluorescence studies, cells (10^6^) were plated on to glass coverslips (30-mm diameter). For immunoblot analysis, cells (10^5^) were plated on to tissue culture dishes (60-mm diameter). For assessment of metabolic/mitochondrial activity by the XTT {sodium 3′-[1-(phenylaminocarbonyl)-3,4-tetrazolium]-bis (4-methoxy-6-nitro) benzene} assays, cells (2×10^4^) were plated on to 96-well tissue culture plates. Cells were grown for 12–24 h in growth medium before further treatment. Cells were exposed to the following various cellular stress inducers: 2 h hyperosmotic stress (0.2, 0.5 or 1 M sorbitol), 2 h arsenite (150, 300 or 500 μM) and 24 h cisplatin [5, 10 or 15 μM *cis*-diammineplatinum (II) dichloride].

### Expression of epitope-tagged p32

HeLa cells were transiently transfected to express pcDNA-p32-Myc-6×His (provided by P. O’Hare, Marie Curie Research Institute, U.K.) or pcDNA-p32(74–282)-Myc-6×His (typically 1 μg plasmid DNA/5×10^5^ cells) using Lipofectamine™ 2000/Opti-MEM (Invitrogen) according to the manufacturer's instructions. After 5 h incubation in a humidified 5% CO_2_ atmosphere at 37°C, Opti-MEM was changed to growth medium and the cells were further incubated for 18 or 24 h.

### Suppression of p32 expression by siRNA

HeLa cells were transiently transfected with 60 nM siRNA [a mixture of 1: 5′-GAAGUUAGCUUUCAGUCCA-3′; 2: 5′-UUUCGUGGUUGAAGUUAUA-3′; 3: 5′-CGCAAGGGCAGAAGGUUGA-3′; 4: 5′-GCGAAAUUAGUGCGGAAAG-3′; or non-specific control siRNA (Thermo Scientific, Dharmacon)], using Lipofectamine™ 2000/Opti-MEM according to the manufacturers’ instructions. Cells with p32 or control siRNA were then maintained in a humidified 5% CO_2_ atmosphere at 37°C in the growth medium for up to 72 h as indicated before processing for immunofluorescence or immunoblotting.

### Immunoblot analysis

Lysates of HeLa cells, following stress and/or transfection, were prepared in RIPA (radioimmunoprecipitation assay) buffer [50 mM Tris/HCl (pH 7.3), 150 mM NaCl, 0.25 mM EDTA (pH 8.0), 1% (v/v) Triton X-100, 1% (w/v) sodium deoxycholate, 0.2% sodium fluoride and 0.1% sodium orthovanadate, supplemented with protease inhibitors (Complete™ Protease Inhibitors, Roche; 1 tablet/50 ml according to the manufacturer's instructions)]. Samples were resolved by SDS/PAGE (12% gel), transferred on to PVDF membranes, blocked with 5% (w/v) non-fat dried skimmed milk powder in TBST (10 mM Tris/HCl, pH 7.5, 150 mM NaCl and 0.1% Tween 20) and immunoblotted using the following commercially available antibodies: mouse monoclonal anti-(p32 N_m_-terminus) (N-terminus of mature p32 protein) antibody [1:1000 dilution, corresponding to residues 76–93, gC1qR (60.11); Abcam], purified mouse anti-[human JNK (c-Jun N-terminal kinase) 1/2] antibody (1:1000 dilution, 554285; BD Biosciences), purified mouse anti-[JNK/SAPK (stress-activated protein kinase) (pT183/pY185)] antibody (1:1000 dilution, 612540; BD Biosciences), rabbit polyclonal anti-PARP [poly(ADP-ribose) polymerase] (H-250) antibody (1:2000 dilution, sc-7150; Santa Cruz Biotechnology), mouse monoclonal anti-Myc (9E10) antibody (1:5000 dilution, sc-40; Santa Cruz Biotechnology), anti-α-tubulin (B-7) antibody (1:5000 dilution, sc-5286; Santa Cruz), anti-Mfn1 and anti-Mfn2 antibodies (1:1000 dilution; Abcam), and anti-Drp-1 antibody (1:1000 dilution; BD Biosciences). Membranes were then washed with TBST, incubated with the appropriate HRP (horseradish peroxidase)-conjugated anti-mouse antibodies in 1% (w/v) non-fat dried skimmed milk powder/TBST and visualized using enhanced chemiluminescence reagents (Thermo Scientific) according to the manufacturer's instructions.

### Immunofluorescence, confocal scanning laser microscopy and image analysis

HeLa cells (untransfected or transfected with plasmids or siRNA, as indicated), were washed with PBS and incubated for 25 min with MitoTracker Red CMXRos (100 nM; Invitrogen). Incubation of cells with 20 μM CCCP (carbonyl cyanide *m*-chlorophenylhydrazone; Sigma–Aldrich) confirmed the mitochondrial membrane potential sensitivity of the MitoTracker Red localization. For co-staining studies, cells were then fixed with 4% (w/v) paraformaldehyde in PBS, permeabilized with 0.2% Triton X-100 in PBS and blocked in 10% (v/v) FBS in PBS [at room temperature (25°C), 20 min]. Primary antibodies were added to sterile-filtered 1% (w/v) BSA in PBS and incubated with the cells (at room temperature, 1 h). The primary antibodies used and their dilutions were as follows: mouse monoclonal anti-(p32 N_m_-terminus) antibody (1:100 dilution; Abcam); mouse monoclonal anti-c-Myc antibody (1:100 dilution, sc-40; Santa Cruz Biotechnology); rabbit anti-calnexin antibody (1:100 dilution, C4731; Sigma–Aldrich); and mouse monoclonal anti-α-tubulin (1:100 dilution, sc-5286, Santa Cruz Biotechnology). Following three rounds of 5-min washes with PBS, Cy2 (carbocyanine)-conjugated anti-mouse or anti-rabbit secondary antibody (1:400 dilution) were added to sterile filtered 1% (w/v) BSA in PBS and incubated for 1 h at room temperature. Nuclei were stained by DAPI (Sigma–Aldrich; 1:15000 dilution in PBS) for 5 min at room temperature before final washes in PBS and mounting on to glass slides with Biomedia Gel Mount (ProSciTech).

Stained cells were examined by confocal laser scanning microscopy using the Leica TCS SP2 imaging system (100×objective). Normal/elongated, fragmented/punctate or fibrillar mitochondrial morphologies were defined by width/length parameters of 1:1, 1:3 and 1:10 respectively. Quantification of each type of mitochondrial morphology upon p32 knockdown or p32 overexpression was assessed by counting 60–80 cells per condition on three independent occasions.

### Sample preparation and TEM (transmission electron microscopy)

Control non-specific or p32 siRNA-treated HeLa cells were pelleted in microfuge tubes with the supernatant discarded and the cell pellets fixed in 2.5% (v/v) glutaraldehyde (Sigma–Aldrich) in PBS for 4 h at room temperature. The cells were then washed with PBS (three rounds of 10 min) each before post-fixing in ice-cold 1% (v/v) osmium tetroxide (Sigma–Aldrich) in PBS for 2 h. After washes with PBS, the cells were dehydrated using successive 15 min ethanol [10, 30, 50, 70, 90, 100 and 100% (v/v)] washes at room temperature. The dehydrated cells were infiltrated with increasing concentrations of LR white resin (ProSciTech) in ethanol consisting of 25, 50, 75 and 100% (v/v) resin for 6 h for each step. After a second change of 100% (v/v) resin, the cells were embedded in fresh resin in gelatin capsules and allowed to gently sink to the bottom to form a loose pellet. The gelatin capsules were capped to exclude air and the resin was polymerized in an oven at 60°C for 24 h.

Embedded cells in blocks were sectioned with a diamond knife on a Leica Ultracut R microtome and ultra-thin sections (90 nm) were collected on to pioloform-coated 100 mesh hexagonal copper grids (ProSciTech). The sections on grids were sequentially stained with saturated uranyl acetate for 10 min and Triple Lead Stain for 5 min [18] and viewed in a Phillips CM120 Biotwin transmission electron microscope at 120 kV.

### High pressure freezing, immunogold labelling and cryo-TEM

Aliquots (2 μl) of control non-specific or p32 siRNA-treated HeLa cell suspensions were pipetted into Leica freezer hats and frozen in a Leica EM PACT2 high pressure freezer. Frozen cell pellets were freeze-substituted in 0.1% uranyl acetate in acetone at −90°C for 48 h and brought to −50°C at 6°C/h. The cell pellets were collected, washed with acetone (three rounds of 30 min) and then infiltrated with increasing concentrations of Lowicryl® HM20 low-temperature resin (Polysciences) in acetone consisting of 25, 50, 75 and 100% (v/v) resin for 8, 12, 8 and 12 h respectively. After embedding in 100% resin in gelatin capsules, the samples were first polymerized under UV light for 48 h at −50°C, brought to room temperature at 6°C/h and then polymerized for another 48 h under UV light at room temperature.

Embedded cells in blocks were sectioned with a diamond knife on a Leica Ultracut R microtome and ultra-thin sections (90 nm) were collected on to pioloform-coated 100 mesh hexagonal gold grids (ProSciTech). The grids were blot dried and incubated with blocking agent [1% (w/v) BSA and 0.1% Tween 20 in PBS) for 30 min. For immunogold labelling, the grids were first incubated on 20 μl droplets of mouse monoclonal anti-(p32 N_m_-terminus) antibody (0.1 μg/ml; Abcam) in blocking agent for 4 h at room temperature, rinsed with the blocking agent (three rounds of 5 min) and then overnight incubated on 20 μl droplets of goat anti-mouse secondary antibody (1:40 diluted in blocking agent) conjugated to 18 nm gold particles at 4°C. Labelled grids were double rinsed with blocking agent PBS, then distilled water before air drying. The immuno-labelled sections on grids were sequentially stained with 2% (w/v) uranyl acetate for 10 min and Triple Lead Stain for 5 min [18] and viewed in a Phillips CM120 Biotwin transmission electron microscope at 120 kV. Images were captured with a Gatan Multiscan 600CW digital camera.

### Measurement of mitochondrial bioenergetics and function

OCR (oxygen consumption rate) and ECAR (extracellular acidification rate), to assess mitochondrial bioenergetics and function, were monitored using the Seahorse XF24 Extracellular Flux Analyser (Seahorse Biosciences) [19]. HeLa cells, transfected with p32-specific siRNA or non-specific control siRNA, were plated in growth medium at 5×10^4^ cells/well the day before analysis. Prior to assay, cells were washed twice with the XF assay buffer (unbuffered DMEM supplemented with 25 mM glucose and 10 mM sodium pyruvate, pH 7.4). Cells were equilibrated in XF buffer in a non-CO_2_ incubator for 60 min before assay. The assay protocol consisted of repeated cycles of mixing (3 min), incubation (2 min) and then measurement (3 min) periods. OCR and ECAR were measured simultaneously. Following basal energetic or respiration measurements, cells were sequentially treated with 1 μM oligomycin (ATP synthase inhibitor), 1 μM FCCP (carbonyl cyanide *p*-trifluoromethoxyphenylhydrazone, proton ionophore) and 1 μM antimycin A (complex III inhibitor) and the changes in respiration were recorded. Mitochondrial function parameters of basal respiration (difference in OCR before treatment of mitochondrial inhibitors and after antimycin A treatment), uncoupled respiration (also known as proton leak, difference in OCR following treatment of oligomycin and antimycin A), spare respiratory capacity (difference in OCR following treatment of FCCP and before any treatment), oxidative ATP turnover (difference in OCR following oligomycin treatment and before any treatment) and maximal respiratory capacity (difference in OCR following treatment of FCCP and antimycin A) were determined [19]. All treatment conditions were analysed in duplicate for at least three independent experiments.

### Measurement of ATP levels

A luminescence assay (ATP Determination Kit, Invitrogen) was used to determine ATP levels. Briefly, before ATP measurements, HeLa cells were pre-treated with p32 or control siRNA for 48 h, and treatment with 50 μM rotenone (Sigma) for 1 h was included as a positive control. Cells were then lysed [ice-cold RIPA buffer supplemented with protease inhibitors (Complete™ Protease Inhibitors, Roche)]. All samples were prepared in triplicate on three independent occasions. Protein concentrations of the lysates were determined and 5 μg of cell lysate was used for the ATP assays according to the manufacturer's instructions. An ATP standard curve was analysed in parallel. Luminescence was measured using a FLUOstar Optima plate reader (BMG Labtech), and values were expressed relative to the control ATP levels measured in the control siRNA-treated cells.

### Cell toxicity assays (XTT and LDH release assays)

A colorimetric assay (Cell Proliferation Kit II, Roche Applied Science) was used to assess the metabolic/mitochondrial activity of cells to cleave XTT to form a soluble orange formazan dye. The XTT assay was carried out according to the manufacturer's instructions using HeLa cells that were pre-treated with p32 or control siRNA for 48 h. A cytotoxicity detection kit (LDH Release Assay, Roche Applied Science) was used to quantitatively assess cell death on the basis of the amount of LDH (lactate dehydrogenase) released into the medium upon damage of the plasma membrane. The LDH assay was conducted according to the manufacturer's instructions also using HeLa cells pre-treated with p32 or control siRNA for 48 h.

### Statistical analyses

Data correspond to the means±S.E.M. (*n*≥3). The unpaired Student’s *t* test was used for comparison of the data, and statistically significant differences are indicated *P<0.05, **P<0.01 and ***P<0.001.

## RESULTS

### p32 is a mitochondrial protein under control and stress conditions

**Figure 1:**
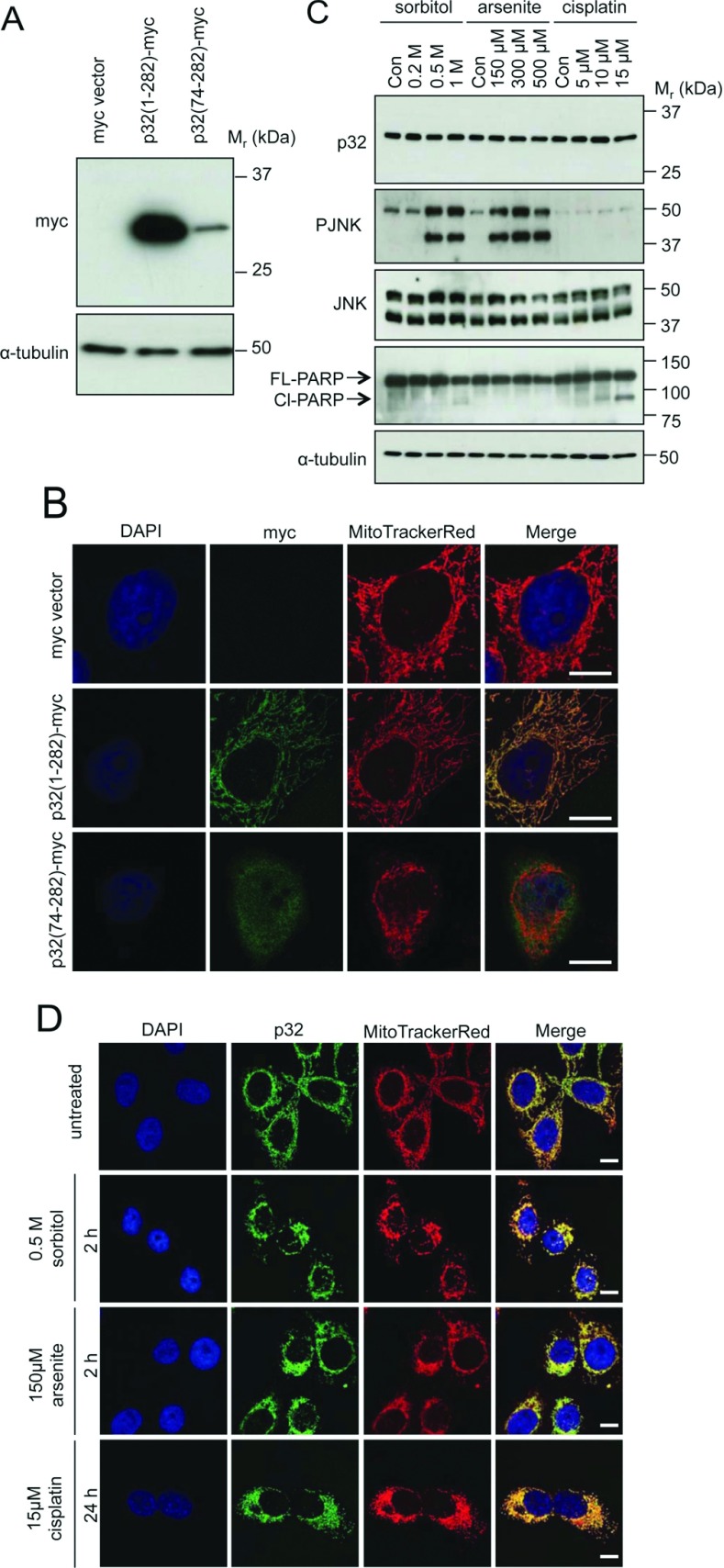
p32 shows mitochondrial localization (**A** and **B**) HeLa cells were transfected with the indicated plasmid constructs. (**A**) After 24 h, cell lysates were immunoblotted with an anti-Myc epitope tag antibody to detect ectopically expressed p32. Immunoblotting for α-tubulin was used as a loading control. (**B**) Cells were stained with MitoTrackerRed (mitochondria), immunostained with anti-Myc antibodies (p32-Myc), then stained with DAPI (nuclei). The merge panels overlay MitoTrackerRed, Myc epitope and DAPI staining. Scale bar is 5 μm. (**C** and **D**) HeLa cells were either left untreated or exposed to stress conditions induced by 2 h treatment with sorbitol (0.5 M), 2 h treatment with arsenite (150 μM) or 24 h treatment with cisplatin (15 μM). (**C**) Cell lysates were immunoblotted as indicated to detect endogenous p32, PJNK, total JNK and full-length (FL) and cleaved (Cl) PARP, with α-tubulin as a loading control. Con, control. Molecular masses are indicated in kDa. (**D**) Cells were stained with MitoTrackerRed (mitochondria), immunostained with anti-p32 antibodies, and then stained with DAPI (nuclei). The merge panels overlay MitoTrackerRed, p32 and DAPI staining. Scale bar is 10 μm. Results are representative of four independent experiments.

Previous reports have detected the p32 protein in various cellular compartments and at the cell surface [4–6]. To define p32 localization under control conditions, we first expressed epitope-tagged p32 protein in HeLa cells. We compared a full-length p32 construct, p32(1–282)–Myc, with an N-terminal truncation, p32(74–282)–Myc, which can mislocalize due to the removal of its mitochondrial localization sequence [20]. Expression of both full-length p32(1–282)–Myc and N-terminal truncated p32(74–282)–Myc constructs was assessed by immunoblotting using an anti-Myc antibody. p32(74–282)–Myc was observed as a ~32 kDa protein similar to the size of the full-length p32(1–282)–Myc construct, but at lower expression levels (Figure 1A). The similarity in size of the detected p32 proteins is consistent with further processing to remove the N-terminus of the full-length p32(1–282) protein and so produce the final 32 kDa product corresponding to p32(74–282) [20].

In parallel experiments, the localization of the ectopically expressed p32 proteins was determined via immunostaining for the Myc epitope tag together with co-staining with MitoTrackerRed dye that accumulates in the mitochondria with intact membrane potential to define mitochondrial morphology and co-localization. Staining of the full-length p32(1–282)–Myc co-localized with that of MitoTrackerRed (Figure 1B). In contrast, the staining of p32(74–282)–Myc did not co-localize with MitoTrackerRed and the staining was diffusely distributed throughout the cell, including both the cytosol and the nucleus identified by DAPI staining (Figure 1B). These results confirm the predominant mitochondrial localization of the p32 protein in HeLa cells under control conditions that appears dependent on an N-terminal mitochondrial targeting sequence.

We extended our analysis to endogenous p32 in HeLa cells. Immunoblotting confirmed the presence of the p32 protein as a ~32 kDa protein, the levels of which did not change during exposure to increasing concentrations of several different stress agents [0.2, 0.5 and 1 M sorbitol (2 h); 150, 300 and 500 μM arsenite (2 h); or 5, 10 and 15 μM cisplatin (24 h)], that were sufficient to affect changes in signalling pathways typified by PJNK (phosphorylated JNK) for sorbitol and arsenite treatment or that resulted in changes in apoptotic signalling pathways mediated by the cleavage of full-length PARP in the case of cisplatin exposure (Figure 1C). As some previous reports have indicated altered p32 localization upon stress [21,22], we also examined the localization of endogenous p32 in HeLa cells. Under control conditions, endogenous p32 co-localized with MitoTrackerRed staining, consistent with a predominantly mitochondrial localization (Figure 1D). Co-localization was similarly observed for p32 and cytochrome *c* staining, again consistent with the dominant mitochondrial localization of p32 protein (M.J. Hu and M.A. Bogoyevitch, unpublished work). Furthermore, we observed mitochondrial staining for endogenous p32 protein across a broad range of cells, including MCF-10, MCF-10A and MCF-7 human epithelial cells, C2C12 mouse skeletal myoblasts, neonatal rat primary cardiac myocytes, GC2 mouse testis germ cells and Vero African green monkey kidney epithelial cells (M.J. Hu, I.H.W. Ng, D.A. Jans and M.A. Bogoyevitch, unpublished work).

In examining the effects of abiotic stress under the conditions verified to alter stress signalling events (Figure 1C), we observed no changes in p32 localization in HeLa cells treated with sorbitol (0.5 M; 2 h), arsenite (150 μM; 2 h) or cisplatin (15 μM; 24 h) (Figure 1D). Thus p32 remains co-localized with MitoTrackerRed under the stress conditions examined. These results agreed with our observations of mitochondrial retention of p32 following stress exposure of COS-1 cells (E.J.H. Boey and M.A. Bogoyevitch, unpublished work).

### Altered p32 levels have an impact on mitochondrial and ER morphologies

**Figure 2:**
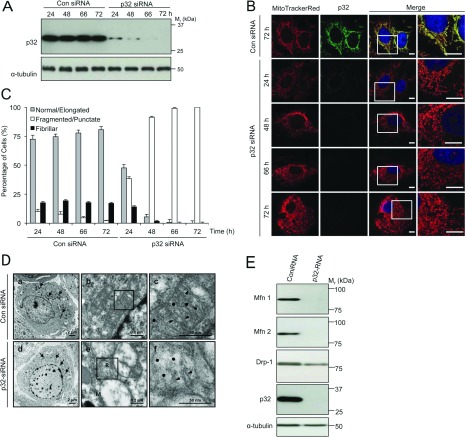
p32 siRNA-mediated knockdown of p32 results in fragmentation of the mitochondrial network HeLa cells were treated with p32-specific siRNA (60 nM) or control siRNA over the time-course indicated. (**A**) Cell lysates were immunoblotted with an anti-p32 antibody to detect endogenous p32. Immunoblotting for α-tubulin was used as a loading control. (**B**) Cells were stained with MitoTrackerRed (mitochondria), immunostained with anti-p32 antibodies, and then stained with DAPI (nuclei). The merge panels overlay MitoTrackerRed, p32 and DAPI staining. The boxed areas in the left merge panels are shown at higher magnification (×2) in the right merge panels. Scale bar is 10 μm. (**C**) Quantification of mitochondrial morphology following reductions in p32 levels. Normal/elongated, fragmented/punctate or fibrillar mitochondrial morphologies were defined by width/length parameters of 1:3, 1:1 and 1:10 respectively. Quantification of each type of mitochondrial morphology following p32 knockdown was assessed for 60–80 cells per condition. Data are means±S.E.M. *P*<0.05 for all mitochondrial morphologies. (**D**) siRNA-treated HeLa cells were subjected to the sample preparation and TEM analysis. Panels a and b highlight individual mitochondrion (shown by arrows). Panels c and f are high magnification (×3) images of the boxed areas in panels b and e, respectively. Panel e highlights mitochondrion with either reduced cristae (shown by R) or with loss of cristae (shown by M). Panels c and f show cristae (black arrowheads) and mitochondrial matrices (black dots). (**E**) Cell lysates from siRNA-treated HeLa were immunoblotted with anti-Mfn1, anti-Mfn2 and anti-Drp-1 antibodies to evaluate levels of these mitochondrial fusion/fission proteins. Immunoblotting for anti-p32 antibody detected endogenous p32 and immunoblotting for α-tubulin was used as a loading control. Results are representative of three independent experiments.

To explore the cellular roles of p32, we used siRNA to reduce endogenous p32 levels. Whereas transfection of HeLa cells with the control non-silencing siRNA did not have an impact on p32 levels, the transfection with p32 siRNA resulted in a substantial (>80%) decrease in p32 levels over 24–72 h as determined by immunoblotting (Figure 2A). Parallel time-course studies that detected the p32 protein by immunostaining and confocal laser scanning microscopy confirmed the loss of the p32 protein in the p32 siRNA-treated cells (Figure 2B). Strikingly, we also observed changes in mitochondrial morphology following p32 depletion. Specifically, mitochondrial morphology following p32 siRNA transfection for 24 h showed a mixed profile of tubular and punctate structures as noted by the MitoTrackerRed staining (Figure 2B). With increasing incubation time with p32 siRNA, smaller, shorter and more punctate mitochondrial morphology was observed (Figure 2B). The continued detection of the mitochondria with MitoTrackerRed was consistent with the retained mitochondrial membrane potential during p32 siRNA treatment, and we confirmed that treatment with the ionophore CCCP disrupted MitoTrackerRed localization under our tested conditions (Supplementary Figure S1 at http://www.biochemj.org/bj/453/bj4530381add.htm). We confirmed that this fragmentation pattern upon gradual p32 depletion was also seen following staining for cytochrome *c* (Supplementary Figure S2 at http://www.biochemj.org/bj/453/bj4530381add.htm). We quantitatively assessed the distribution of normal/elongated, fragmented/punctate and fibrillar mitochondria when p32 expression levels were lowered. These results highlight the significant increase in fragmented/punctate mitochondria upon p32 siRNA transfection and loss of p32 levels (Figure 2C, *P*<0.05 for all mitochondrial morphologies).

To define the changes in mitochondrial morphology due to p32 depletion with higher resolution, the ultrastructure of mitochondria in p32 siRNA-treated cells was analysed by TEM. Consistent with the results obtained using confocal microscopy (Figures 2B and 2C), the p32 siRNA-treated cells contained predominantly small spherical mitochondria, compared with the mainly normal elongated mitochondria in control cells (Figure 2D). Whereas the TEM analysis revealed no detectable changes in the overall distribution of mitochondria in control and p32 siRNA-treated cells (Figure 2D, panels a and d, arrows indicate mitochondria), changes in the mitochondrial inner membrane and matrix were observed in p32 siRNA-treated cells (Figure 2D, panels e and f). Specifically, compared with lamellar crista structure in mitochondria of the control cells (Figure 2D, panels b and c, arrowheads for cristae), the majority of the mitochondria in p32 siRNA-treated cells were found to be either completely devoid of cristae (Figure 2D, mitochondrion labelled as M in panel e) or with significantly reduced crista content along with its shortening from lamellar to tubular profiles (Figure 2D, mitochondrion labelled as R in panel e, arrowheads for cristae in panel f). In addition, the matrices of many of the small mitochondria in p32 siRNA-treated cells appeared less electron-dense than those in the control cells (Figure 2D, panels c and f, labelled as dots). To explore the involvement of mitochondrial fusion and fission regulatory proteins, we examined the expression of Mfn1, Mfn2 and mitochondrial Drp-1. Although all proteins were clearly detected under control conditions, the p32 siRNA treatment resulted in a loss of detectable Mfn1 and Mfn2 and decreased Drp-1 levels (Figure 2E). Taken together, the results show that the loss of p32 resulted in fragmentation of mitochondria with alterations in crista structure and matrix density.

**Figure 3:**
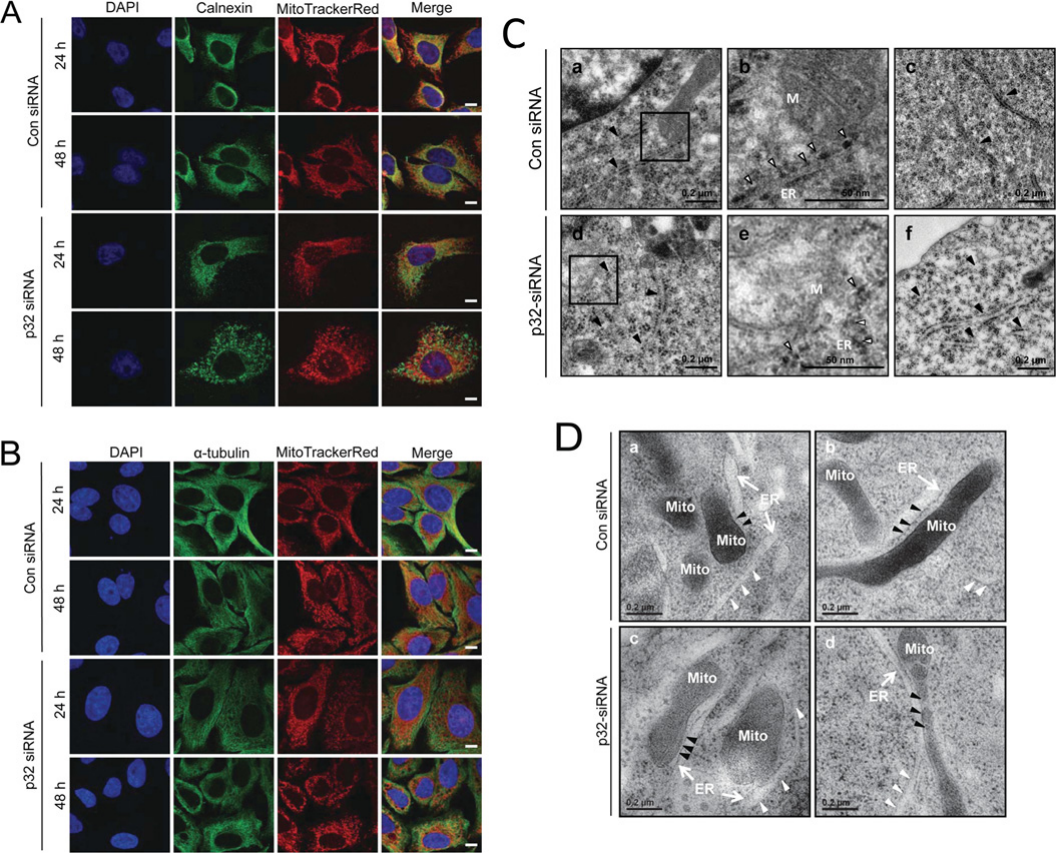
Effect of p32 siRNA-mediated knockdown of p32 on organization of the ER and microtubules HeLa cells were pre-treated with p32-specific siRNA (60 nM) or control siRNA for 48 h. (**A**) Cells were stained with MitoTrackerRed (mitochondria), immunostained with anti-calnexin antibodies (ER), and then stained with DAPI (nuclei). The Merge panels overlay MitoTrackerRed, calnexin and DAPI staining. (**B**) Cells were stained with MitoTrackerRed (mitochondria), immunostained with anti-α-tubulin antibodies, then stained with DAPI (nuclei). The merge panels overlay MitoTrackerRed, α-tubulin (microtubules) and DAPI staining. Scale bar is 10 μm. (**C**) siRNA-treated cells were immediately subject to the sample preparation and TEM analysis. TEM images show ER with arrowheads, associated ribosomes with hollow arrowheads and mitochondrion denoted M. Panels b and e are high magnification (×3) images of the boxed areas in panels a and d respectively. Results are representative of three independent experiments. (**D**) Control and p32 siRNA-treated HeLa cells were subjected to high pressure freezing, immunogold labelling and cryo-TEM analysis. Individual mitochondrion and ER were highlighted by Mito and arrows respectively. Black arrowheads indicate ER–mitochondria contact points, and white arrowheads indicate ER not in contact with mitochondria. Results were representative images taken from a pool of 25 images for each condition.

As there is an increasing appreciation for the close physical and functional association between mitochondria and ER [23–25], we examined the impact of p32 silencing on ER morphology. The reticular staining pattern of calnexin, an ER chaperone protein, showed a less elongated network following 24 h of p32 siRNA transfection (Figure 3A). After 48 h of p32 siRNA, significant changes in the ER morphology as indicated by calnexin staining were observed with a higher number of punctate ER structures compared with tubule structures (Figure 3A). In parallel experiments, p32 siRNA had no effect on microtubule organization as assessed by α-tubulin staining (Figure 3B).

TEM analyses were also performed in p32 siRNA-treated HeLa cells to examine more closely the change of ER morphology at the ultrastructural level. Consistent with the results obtained using confocal microscopy (Figure 3A), p32 depletion resulted in fragmented or less elongated ER structures (Figure 3C, arrowheads). Ribosomal dissociation (Figure 3C, panels b and e, hollow arrowheads) was also observed for ER close to the mitochondria (Figure 3C, panels b and e, denoted by M) as well as for ER distributed throughout the cytoplasm (Figure 3C, panels c and f). These results thus indicate that lowering p32 protein levels can lead to more punctate mitochondrial and ER structures with no detectable changes in the cellular architecture as assessed by microtubule organization. We further used high pressure freezing and cryo-TEM analysis to examine the potential impact of p32 knockdown on ER–mitochondria contact points. Our immunogold labelling of p32 protein confirmed its mitochondrial localization (Figure 3D, panels a and b) and the effective knockdown of p32 protein in the p32 siRNA-treated samples (Figure 3D, panels c and d). No detectable changes were observed in an evaluation of the ER–mitochondria contact points across a pool of 25 images, thus suggesting that p32 siRNA-mediated knockdown of p32 did not cause major differences in ER–mitochondria contact points.

**Figure 4:**
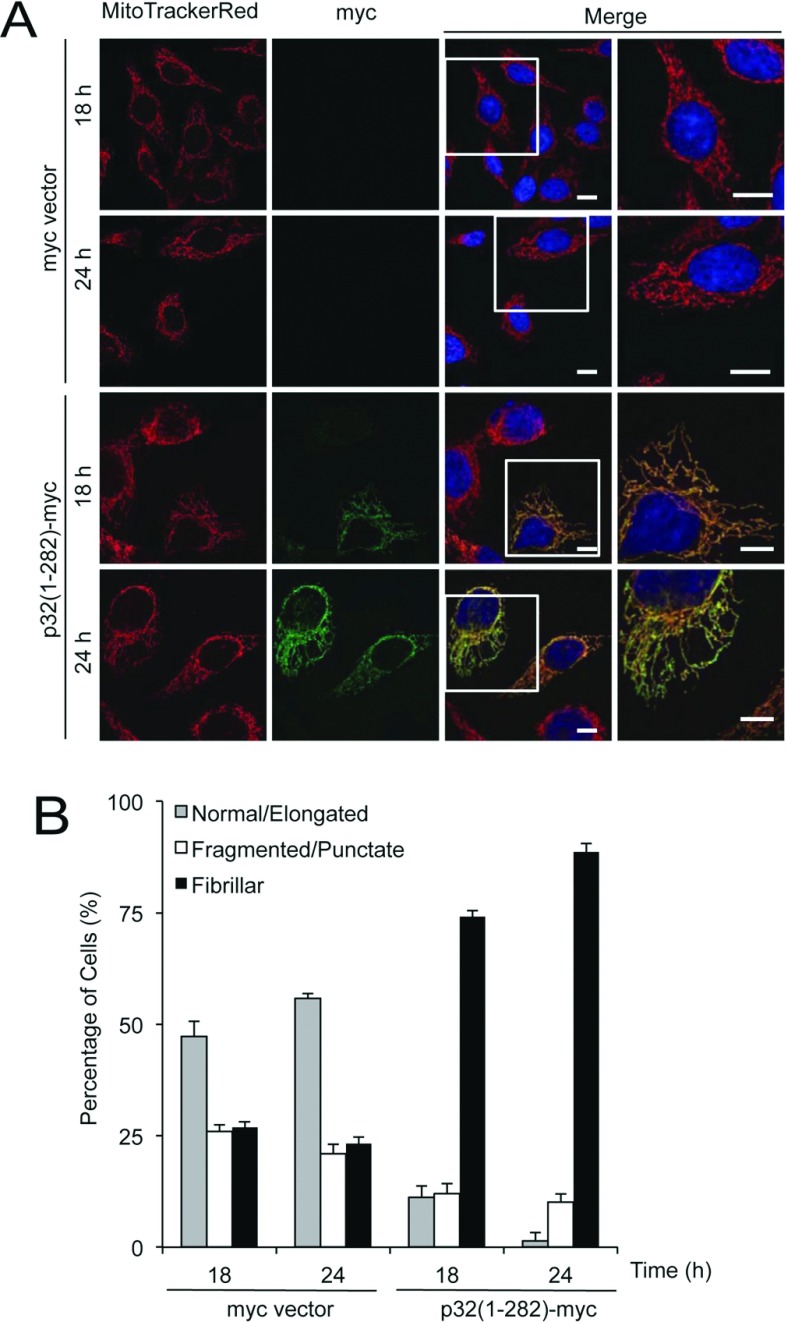
Overexpression of p32 results in fibrillar mitochondrial morphology (**A**) HeLa cells were transfected with Myc vector or p32(1–282)–Myc, stained with MitoTrackerRed (mitochondria) and immunostained with anti-Myc antibodies (p32-Myc) at 18 or 24 h post-transfection as indicated. The merge panels overlay MitoTrackerRed, and Myc epitope staining. The boxed areas in the left merge panels are shown at higher magnification (×2) in the right merge panels. Scale bar is 10 μm. (**B**) Quantification of mitochondrial morphology following increase in p32 levels. Normal/elongated, fragmented/punctate or fibrillar mitochondrial morphologies were defined by width/length parameters of 1:3, 1:1 and 1:10 respectively. Quantification of each type of mitochondrial morphology following p32 overexpression was assessed for 60–80 cells per condition. Data are means±S.E.M. *P*<0.05 for all mitochondrial morphologies. Results are representative of four independent experiments.

To explore further the impact of altered p32 levels on mitochondrial morphology, we evaluated the consequences of the converse situation of increased expression of p32 through transient transfection of the full-length p32(1–282)–Myc construct for 18 or 24 h. Immunostaining for the Myc epitope tag again showed the co-localization of overexpressed p32 with the mitochondria (Figure 4A), which further reinforced the results presented in Figure 1. Significantly, when the mitochondrial morphologies were examined in the transfected and non-transfected cells, a fibrillar thread-like mitochondrial network was observed following the p32 overexpression for 18 or 24 h (Figure 4A). We quantitatively assessed the distribution of normal/elongated, fragmented/punctate and fibrillar mitochondria when p32 expression levels were elevated and fibrillar mitochondria were shown to increase (Figure 4B, *P*<0.05 for all mitochondrial morphologies). Taken together, the results indicate that alteration of p32 levels can significantly affect mitochondrial morphology under control conditions.

### Bioenergetic analysis and assessment of mitochondrial function

**Figure 5:**
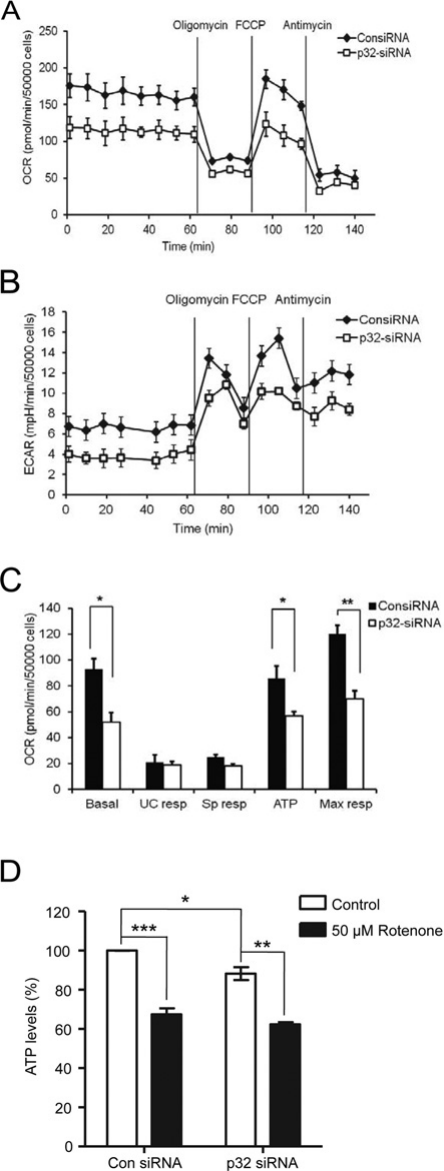
p32 depletion impairs cellular bioenergetics characterized by defective basal cellular respiration and oxidative ATP turnover and lower ATP levels HeLa cells were treated with p32-specific siRNA (60 nM) or non-specific control siRNA (consiRNA) (60 nM) for 48 h. (**A**–**C**) Mitochondrial bioenergetic analysis was performed using the Seahorse extracellular flux analyser XF40 upon sequential additions of oligomycin (1 μM), FCCP (1 μM) and antimycin A (1 μM). (**A**) OCR as an indication of mitochondrial respiration and (**B**) ECAR as a function of glycolytic capacity were simultaneously measured in real time. (**C**) Mitochondrial function parameters of basal respiration (Basal), uncoupled respiration (UC resp, alternatively known as proton leak), spare respiratory capacity (Sp resp), oxidative ATP turnover (ATP) and maximal respiratory capacity (Max resp) were determined. Data were average values pooled from at least three independent experiments with two replicates each. Error bars represent S.E.M. **P<0.01 and *P<0.05. (**D**) ATP levels were determined by a luminescence assay. HeLa cells were pre-treated with p32 or control siRNA (48 h) before treatment with rotenone (50 μM, 1 h) as a positive control. Luminescence values were expressed relative to the control ATP levels measured in the control siRNA-treated control cells. Data represent the means±S.E.M. ***P<0.001 and **P<0.01 and *P<0.05.

To examine a relationship between p32-mediated changes in mitochondrial morphology and mitochondrial function/energy status, the respiratory and glycolytic profiles of the non-specific control and p32 siRNA-treated HeLa cells were measured. In particular, OCR, as an indicator of mitochondrial respiration, and ECAR, as a function of glycolytic lactate production, by non-specific control and p32 siRNA-treated cells were simultaneously measured using a Seahorse extracellular flux analyser [19]. Basal energetic levels were established before sequential exposure of compounds modulating mitochondrial function, i.e. oligomycin (ATP synthase inhibitor), FCCP (proton ionophore) and antimycin A (mitochondrial complex III inhibitor). For p32 siRNA-treated cells, decreased basal OCR (Figure 5A) and basal ECAR (Figure 5B) were observed when compared with the non-specific control siRNA-treated cells.

To examine further the bioenergetic capabilities of the cells, oxidative ATP turnover, uncoupled respiration (alternatively known as proton leak), spare and maximum respiratory capacities were analysed. Compared with control siRNA-treated cells, p32 siRNA-treated cells showed a significant reduction in the ATP turnover rate and maximum respiration in addition to decreased basal respiration (Figure 5C). Depletion of p32 did not significantly affect uncoupled and spare respiratory capacities (Figure 5C). Thus the analysis of mitochondrial function showed the defect in basal respiration was largely attributed to a reduction in mitochondrial ATP turnover, suggesting that the inhibition of ATP turnover is the primary bioenergetic parameter modulated by p32 depletion. We therefore extended our analysis to the detection of ATP levels in these cells. We included treatment with rotenone as a positive control, showing its ability to decrease ATP levels significantly in control siRNA-treated cells (Figure 5D). For p32 siRNA-treated cells, we noted a small, but statistically significant decrease in measured ATP levels and again these were further decreased in the presence of rotenone (Figure 5D). Thus depletion of p32 did lead to lowered intracellular ATP levels.

### p32 silencing differentially affects cell viability following exposure to different stresses

**Figure 6:**
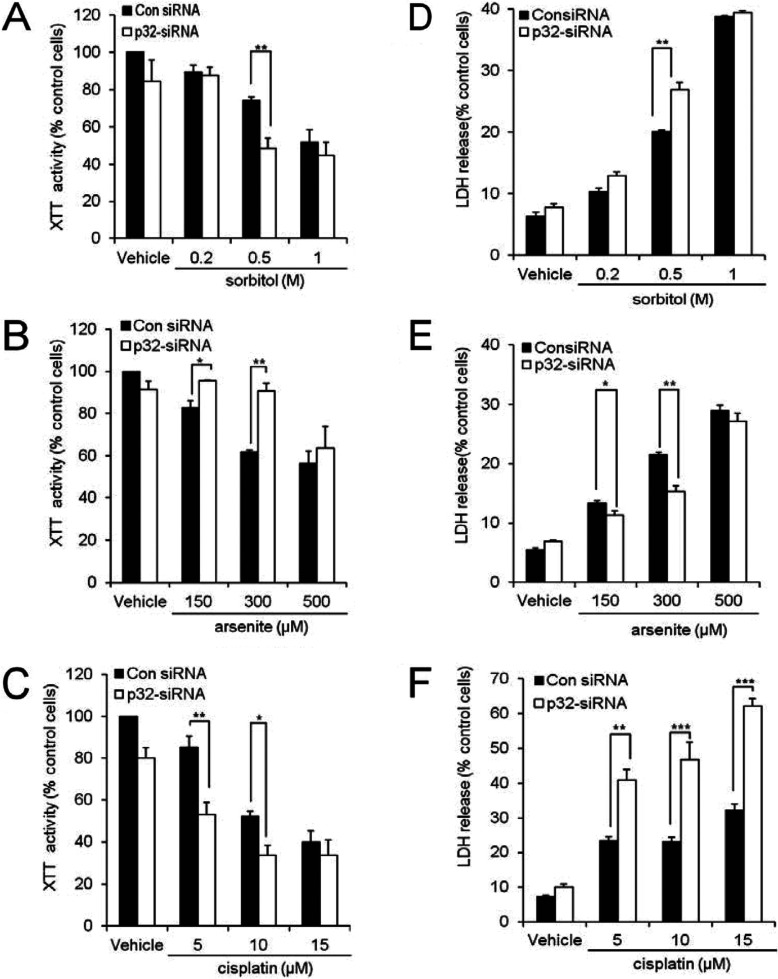
Effect of p32 siRNA-mediated knockdown of p32 on stress-induced changes in XTT activity and LDH release HeLa cells were treated with p32-specific siRNA (60 nM) or control siRNA (consiRNA) for 48 h then exposed to (**A** and **D**) sorbitol (0.2, 0.5 and 1 M) for 2 h, (**B** and **E**) arsenite (150, 300 and 500 μM) for 2 h or (**C** and **F**) cisplatin (5, 10 and 15 μM) for 24 h. (**A**–**C**) Cells were then incubated with the XTT reaction mixture for 4 h and the XTT activity was determined as a percentage of the control cell activity. (**D**–**F**) The cultured media of cells treated with each stress were incubated with the LDH reaction mixture for 30 min. Optical densities were determined using an ELISA microplate reader (test wavelength 490 nm, reference wavelength 655 nm) and adjusted for the background as determined by treatment- and cell-free media. Each experiment was repeated on three independent occasions with similar results. Data are the means±S.E.M. ***P<0.001, **P<0.01 and *P<0.05.

Changes in mitochondrial morphology have been linked to energy metabolism and cell death, but the detailed mechanisms linking these changes remain largely undefined [12–14]. The contrasting mitochondrial morphologies upon increased and lowered p32 expression levels prompted us to investigate the impact of endogenous p32 protein on cell survival following exposure to different stress conditions. First, we assessed how lowered p32 expression levels affect cell responses under control conditions and in response to abiotic stresses by using the XTT colorimetric assay for cytotoxicity. Under control conditions, transfection with the p32 siRNA does not significantly decrease the XTT activity of HeLa cells (Figures 6A–6C). For control siRNA-treated cells, sorbitol (6 h), arsenite (6 h) or cisplatin (28 h) at increasing doses decreased XTT activity (Figures 6A–6C). For sorbitol exposure (0.5 M) or cisplatin exposure (5 or 10 μM), the p32 siRNA treatment enhanced the loss of XTT activity (Figures 6A and 6C respectively). Conversely, p32 siRNA treatment resulted in a significant effect on maintenance of XTT activity following exposure to arsenite at 150 or 300 μM, but not 500 μM arsenite (Figure 6B).

To confirm the impact of altered p32 levels on the survival of cells following different stress conditions, we assessed the release of the cytosolic enzyme, LDH, into the culture medium. Under control conditions, transfection with the p32 siRNA resulted in an increasing trend in LDH release, indicating lowered cell viability following p32 silencing (Figures 6D–6F). For control siRNA-treated cells, sorbitol (2 h), arsenite (2 h) or cisplatin (24 h) at increasing doses enhanced LDH release (Figures 6D–6F). For sorbitol (0.5 M) exposure or cisplatin exposure (5 or 10 μM), the p32 siRNA treatment further accentuated the increase in LDH release, indicative of increased cell death (Figures 6D and 6F respectively). Conversely, p32 siRNA treatment reduced the LDH release following exposure to arsenite (150 or 300 μM), suggesting that the loss of p32 protects against arsenite-induced cell death (Figure 6E). Thus the siRNA-induced loss of p32 has a negative impact on the survival of cells challenged with sorbitol or cisplatin, but contributes to cell survival when cells are exposed to arsenite.

## DISCUSSION

This is the first demonstration of the requirement for the p32 protein in the maintenance of the morphology of both the mitochondrial and ER networks. It is increasingly appreciated that mitochondria exist as dynamic networks that often change morphology according to the changing requirements of the cell. The control of mitochondrial morphology is considered to be a result of the complex balance of activity of several proteins that affect mitochondrial fusion [including Mfn1 and Mfn2 proteins, Opa1, and scaffold proteins that regulate Opa1 levels namely prohibitin 2 and SLP-2 (stomatin-like protein-2)] and mitochondrial fission [including MTP18 (mitochondrial protein 18 kDa) and Drp-1, and mitochondrial outer membrane-associated proteins that recruit Drp-1 to mitochondria such as mitochondrial Fis1 (fission 1) protein and GDAP1 (ganglioside-induced differentiation-associated protein 1)] [14,15,17]. Although mitochondrial fusion is considered to be critical for ensuring homogeneity of the mitochondrial population, mitochondrial fission is critical for the mitophagic clearing of old/damaged mitochondria as well as creating new mitochondria during growth and cell division [14,26]. Perturbations to mitochondrial remodelling via altered fission and fusion events have been implicated in a wide variety of human pathologies, typically cancer and age-related diseases [27–31]. Therefore an improved understanding of the proteins controlling mitochondrial morphology, fission and fusion, will be critical for improving approaches to manipulate these processes as part of therapeutic strategies to enhance or maintain mitochondrial integrity and function.

That levels of the mitochondrial matrix protein p32 strongly affect mitochondrial morphology, where increased levels drive a more fibrillar mitochondrial morphology (Figure 4) and decreased levels drive a fragmented/punctate morphology (Figure 2), suggests that p32 may directly modulate mitochondrial fission/fusion machinery. To date, there has been a range of potential p32-interacting partners identified [4,8,9]. However, no known regulators of mitochondrial morphology have been identified as direct interacting partners for p32. The enhanced mitochondrial fragmentation we observed following p32 silencing would be consistent with up-regulated mitochondrial fission and/or a loss of mitochondrial fusion events and this is consistent with the loss of detectable levels of Mfn1 and Mfn2 proteins that we observed following p32 siRNA treatment (Figure 2E).

The loss of the p32 protein alters metabolism in yeast [1]; in the context of tumour cells, p32 siRNA has been associated with a switch between glycolysis and oxidative phosphorylation [10]. In the present study, we have shown the impact of the knockdown of p32 in attenuating oxidative phosphorylation capacity typified by reduced basal respiration, maximum respiratory capacity ATP turnover and ATP levels (Figure 5). However, the ECAR showed a tendency of reduced glycolysis with lowered p32 protein levels. Furthermore, p32 knockdown resulted in lowered mitochondrial activity as assessed by XTT assays, i.e. under control conditions, p32 knockdown resulted in lower XTT activities, albeit that these changes were small (<20% decreases in activities of control cells in Figures 6A–6C). Strikingly, the presence of different stresses induced by the presence of cisplatin or hyperosmotic stress induced by high concentrations of sorbitol could also decrease XTT activity, with these decreases being more pronounced in the presence of p32 siRNA. In contrast, the p32 siRNA treatment was able to prevent the loss in XTT activity following exposure to 150 or 300 μM arsenite. The mechanisms underlying this differential response clearly require further exploration, particularly with a focus on the underlying initiating stress and signalling events and how these can then trigger the subsequent cellular changes.

Mitochondria are also critical contributors to apoptotic cell death in response to a range of stress stimuli. In addition to outer membrane permeabilization, extensive mitochondrial fragmentation via fission events, together with remodelling of the mitochondrial inner membrane cristae, are hallmarks of cell death [32,33]. Proapoptotic proteins such as Bax and Bak have been shown to influence mitochondrial morphology through direct association with Mfn1/2 [34]. A number of fission and fusion proteins, including Mfn1, Mfn2, Opa1 and Drp-1, have been directly implicated in the regulation of apoptosis [32,33], consistent with a close relationship between mitochondrial morphology and cell death processes. The results of the present study for cell death by release of LDH activity from p32 siRNA-treated cells during stress conditions were consistent with the results from our XTT assays, i.e. loss of p32 enhances cell death in response to cisplatin or sorbitol, but contributes to cell survival in response to 150 or 300 μM arsenite (Figures 6D–6F). Intriguingly, these different responses are in the context of fragmented mitochondrial morphologies that have been most commonly associated with an enhanced cell death response [35–37]. Clearly the biochemical networks directed by p32 that regulate mitochondrial morphology, metabolic activities and cell death pathways warrant further studies.

Lastly, there has been an increased appreciation in recent years of the close physical and functional association between mitochondria and ER [23–25]. The p32 N-terminus sequence (amino acids 1–73) has been assigned previously as a mitochondrial targeting sequence [20], and in line with these observations we have consistently observed mitochondrial localization of p32 across a range of cell types and under a range of different stress conditions (Figure 1). We used calnexin staining and electron microscopic analysis to evaluate the morphology and structure of ER in p32-deficient cells. The striking changes in ER, with enhanced punctate ER and ribosomal dissociation when p32 levels were lowered (Figure 3), again emphasize the important relationships between the mitochondria and ER and are consistent with the co-ordination of their functions [23–25]. The ER typically forms a network of sheet-like cisternae (the nuclear envelope ER) interconnected with tubules that spread throughout the cytoplasm (the peripheral ER). Similar to the mitochondrial network, the ER is highly dynamic and its tubules can constantly fuse and divide to drive morphological changes [38,39]. The ER and mitochondria have been known to exhibit tightly coupled dynamics, with 5–20% MAMs (mitochondria-associated membranes) in close contact with the ER [23–25]. The close relationship of the ER and mitochondria extends to their exchange of lipids, co-regulation of calcium flux and a co-ordination of their activities in cell survival and death [23,40]. Although we could not detect changes in the mitochondrial–ER contacts using a cryo-TEM approach (Figure 3D), consideration of these more complex inter-organelle relationships will be critical for improved understanding of the impact that p32 has on overall cellular activities. This will be particularly important in interpretation of recent studies including the implication of p32 in a diversity of biological activities including neuronal synaptic transmission [41], responses to viral infections [7] and cell migration [11]. Taken together, our studies highlight the critical contributions of the p32 protein to the morphologies and structures of mitochondria and ER under normal cellular conditions, and demonstrate important roles for the p32 protein in cellular metabolism and responses to different stresses.

## Online data

Supplementary data
