# Supplementary material for: p32 protein levels are integral to mitochondrial and endoplasmic reticulum morphology, cell metabolism and survival
Source: Biochem J. 2013 Jul 12;453(Pt 3):381–91. doi: 10.1042/BJ20121829 (PMC3727215; doi:10.1042/BJ20121829)
Supplement: Supplementary data [file bj4530381add.pdf]

BJ Cell

Biochem. J. (2013) **453**, 381–391 (Printed in Great Britain) doi:10.1042/BJ20121829

online data

SUPPLEMENTARY ONLINE DATA

p32 protein levels are integral to mitochondrial and endoplasmic reticulum morphology, cell metabolism and survival

MengJie HU*, Simon A. CRAWFORD†, Darren C. HENSTRIDGE‡, Ivan H. W. NG*§, Esther J. H. BOEY*[1], Yuekang XU*, Mark A. FEBBRAIO‡, David A. JANS§ and Marie A. BOGOYEVITCH*[2]

*Department of Biochemistry and Molecular Biology, Bio21 Molecular Science and Biotechnology Institute, University of Melbourne, Melbourne, VIC 3010, Australia, †School of Botany, University of Melbourne, Melbourne, VIC 3010, Australia, ‡Cellular and Molecular Metabolism Laboratory, Baker IDI Heart and Diabetes Institute, Melbourne, VIC 3004, Australia, and §Department of Biochemistry and Molecular Biology, Monash University, Melbourne, VIC 3800, Australia

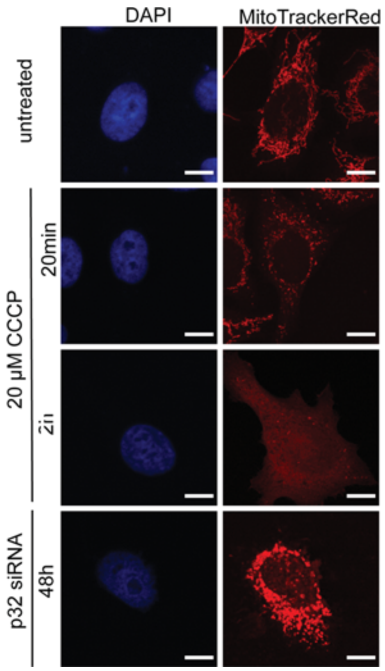

Figure S1 CCCP, but not p32-siRNA, disrupts the mitochondrial-membrane-potential-dependent staining by MitoTrackerRed

HeLa cells were treated with p32-specific siRNA (60 nM) for 48 h or 20 μM CCCP for the times indicated. Cells were stained with MitoTrackerRed (mitochondria), and then stained with DAPI (nuclei). Scale bar is 10 μm.

Biochemical Journal www.biochemj.org

[1] Present address: Cardiac Hypertrophy Laboratory, Baker IDI Heart and Diabetes Institute, Melbourne, VIC 3004, Australia
[2] To whom correspondence should be addressed (email marieb@unimelb.edu.au).

© 2013 The Author(s)
The author(s) has paid for this article to be freely available under the terms of the Creative Commons Attribution Licence (CC-BY) (http://creativecommons.org/licenses/by/3.0/) which permits unrestricted use, distribution and reproduction in any medium, provided the original work is properly cited.

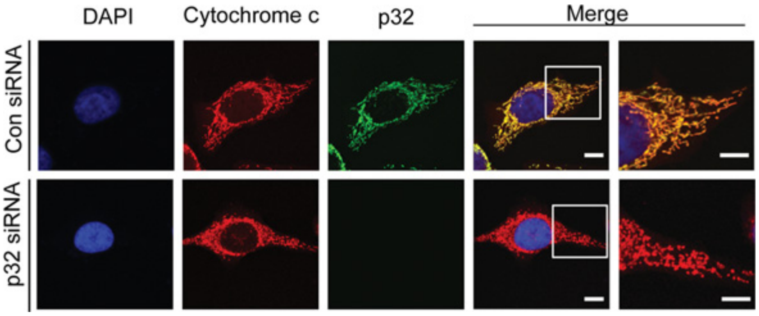

Figure S2 Staining for cytochrome *c* confirms mitochondrial puncta following p32-siRNA treatment

HeLa cells were treated with control or p32-specific siRNA (60 nM) for 48 h. Cells were stained with anti-cytochrome *c* (mitochondria) and anti-p32 antibodies and then stained with DAPI (nuclei). The merge panels overlay cytochrome *c* and p32 staining. The boxed areas in the left merge panels are shown at higher magnification (×2.75) in the right merge panels. Scale bar is 10 μm.

Received 10 December 2012/17 May 2013; accepted 22 May 2013
Published as BJ Immediate Publication 22 May 2013, doi:10.1042/BJ20121829

© 2013 The Author(s)
The author(s) has paid for this article to be freely available under the terms of the Creative Commons Attribution Licence (CC-BY) (http://creativecommons.org/licenses/by/3.0/) which permits unrestricted use, distribution and reproduction in any medium, provided the original work is properly cited.
